# Dataset for residual stress measurements via neutron diffraction of thermally sprayed Inconel 625 coating on 304 stainless steel

**DOI:** 10.1016/j.dib.2024.110041

**Published:** 2024-01-08

**Authors:** Oluseyi Philip Oladijo, Vladimir Luzin, Michael Oluwatosin Bodunrin, Tien-Chen Jen, Resego Phiri

**Affiliations:** a Department of Chemical, Materials & Metallurgical Engineering, Botswana International University of Science and Technology, Palapye, Botswana; b Department of Mechanical Engineering Science, University of Johannesburg, Auckland Park Kingsway Campus, 2006, South Africa; c Australian Centre of Neutron Scattering, Australia Nuclear Science and Technology Organisation, Lucas Heights, NSW 2234, Australia; d School of Chemical and Metallurgical Engineering, University of Witwatersrand, South Africa

**Keywords:** Residual stress properties, Inconel 625, Coating thickness, High-velocity oxy-fuel (HVOF), Neutron diffraction

## Abstract

Fundamental understanding of factors and mechanisms controlling the residual stress formation in material coatings is critical for selection of optimum synthesis and deposition parameters. This article contains data from the investigation of the residual stress properties of Inconel 625 coating measured at different coating thicknesses, 250 µm,300 µm, 350 µm and 400 µm, deposited on 304 stainless steel (SS) substrate using high-velocity oxy-fuel (HVOF) spraying technique. The neutron diffraction technique was employed to measure the residual stresses of the coated specimen. Data provided provides insights into the influence of coating thickness on the residual stress of the material and therefore on the overall mechanical performance and applicability of the component.

Specifications Table

**Table:** 

Subject	Surfaces, Coatings and Films
Specific subject area	Surface coatings and residual stress determination. Involves understanding the techniques and methodologies used to deposit coatings onto surfaces and then measuring the residual stresses that develop during the coating process.
Data format	Raw, Analyzed, Filtered
Type of data	Table, Graph, Figure
Data collection	Inconel 625 coating was thermally deposited using the Sulzer Metco HVOF hybrid DJ-2600 system at Thermaspray Company, South Africa. Residual stress measurements were conducted using Neutron diffraction at the ANSTO OPAL reactor neutron facility using the KOWARI diffractometer.
Data source location	Thermaspray company, Olifantsfontein, South Africa Australia Nuclear Science and Technology Organisation, Lucas Heights, NSW 2234, Australia Botswana International University of Science and Technology, Palapye, Botswana
Data accessibility	Repository name: Mendeley data repository Data identification number: 10.17632/8nz52pmttw.1 Direct URL to data: https://data.mendeley.com/datasets/8nz52pmttw/1
Related research article	O. P. Oladijo, V. Luzin, N. B. Maledi, K. Setswalo, T. P. Ntsoane, and H. Abe, “Residual Stress and Wear Resistance of HVOF Inconel 625 Coating on SS304 Steel Substrate,”2020. https://doi.org/10.1007/s11666-020-01066-x

## 1 Value of the Data

• Controlling the build-up of residual stresses during thermal spray deposition is a crucial aspect in the performance of the coat and therefore very important for ensuring component reliability [1,2]. The data given provides information on the influence of coating thickness on the residual stress behavior of material.
• The data can be used for modelling parameters in order to estimate optimum deposition parameters and thicknesses to be implemented during design of experiments. Furthermore, the data can assist in coating system property manipulation for various material applications.
• The data can be used as an educational tool to demonstrate to learners/students how to calculate residual stresses of metals and other materials from raw neutron diffraction measurements.
• Researchers can use this data to compare with other measurements from other techniques for equipment/methodological assessment.

## 2 Data Description

**Fig. 1:**
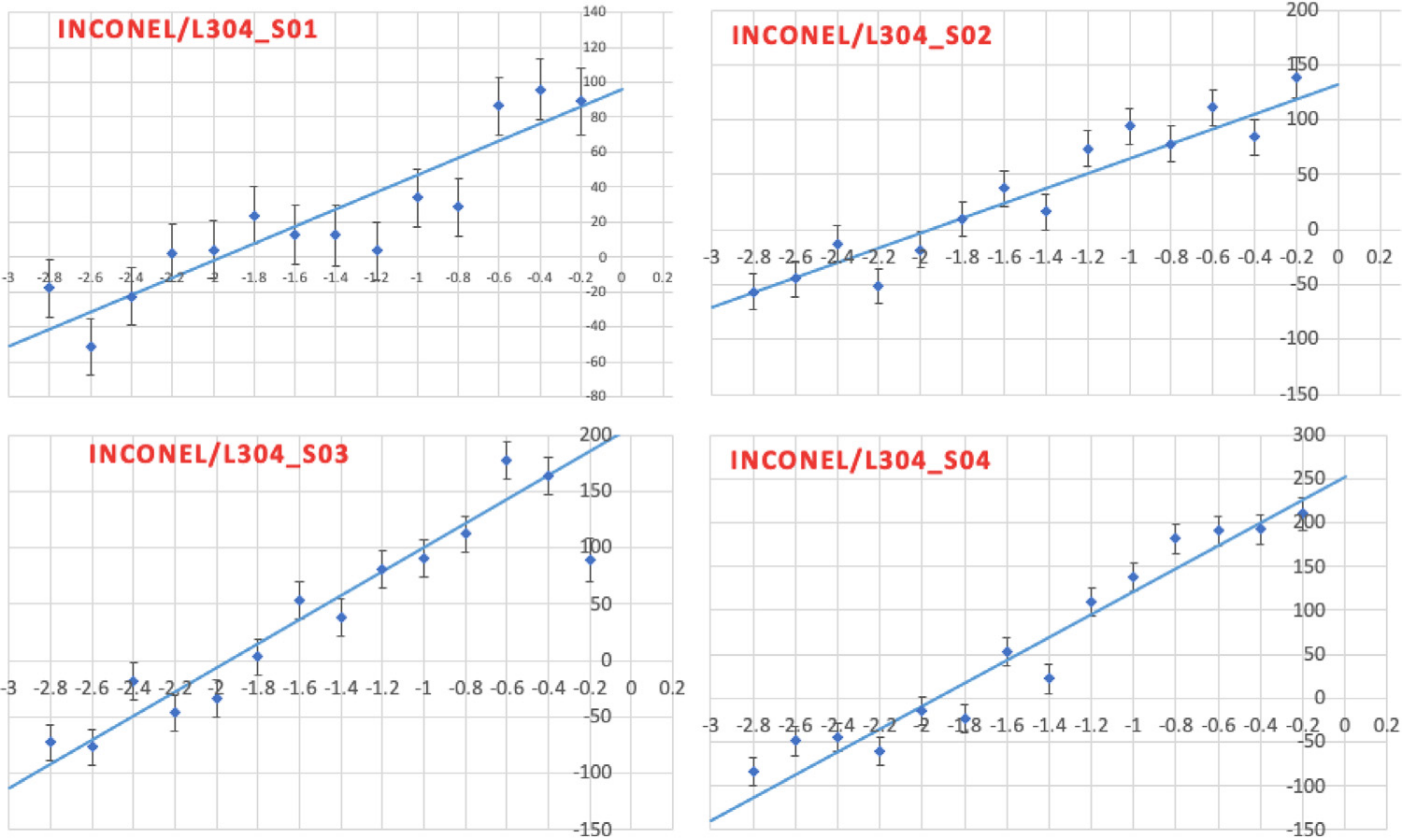
Through-thickness in-plane residual stress profiles in Inconel 625 coatings of varying thickness; 250 µm (S01), 300 µm (S02), 400 µm (S03) and 500 µm (S04) deposited on 304 SS, as measured using neutron diffraction [3].

Neutron diffraction was conducted on four samples with varying Inconel 625 coating thicknesses deposited on 304 stainless steels; sample 1 (S01) with a thickness of 250 µm, sample 2 (S02) with coating thickness of 300 µm, sample 3 (S03) with a coating thickness of 400 µm and finally sample 4 (S04) with a coating thickness of 500 µm. The dataset consists of one figure (Fig. 1) containing four graphs, which were generated through an analysis of raw data. These graphs depict the depth-resolved through-thickness in-plane residual stress profile in an Inconel 625 coating with varying thicknesses (250 µm, 300 µm, 400 µm, and 500 µm). More data on the measurement of residual stresses on Inconel coated samples are available in the repository file (Inconel_HVOF.xlsx), this includes the raw, unfiltered data collected from the neutron equipment. The xlsx file comprises of multiple sheets each containing data of high significance in residual stress measurements.

From the summary sheet in the xlsx file, the thickness of the substrate materials, the thickness of the coatings and experimental conditions for each sample are provided in terms of the wavelength, take-off angle, mono reflection, Braggs angle, Braggs reflection and the gauge volume. In the results section of the summary sheet, the in-plane residual stress profiles in Inconel 625 coatings are illustrated for each of the samples. Mean stress values are given and standard deviations in each sample is presented as error bars. The excel sheet designated S0, S1,S2, S3 and S4, shows data for different crystallographic directions for each Inconel coating 625 coated samples respectively. These directions are used in the description of anisotropic properties and are essential when interpreting residual stress data. Radial Direction (RD) is a direction that extends outward from the centre of the material, typically perpendicular to the axis of rotation or growth. Normal Direction (ND) is a direction perpendicular to the surface of the material. It is often used as a reference direction for describing stress states in thin films or coatings. In many cases, the ND is aligned with the thickness direction of a sample. Tangential Direction (TD) is a direction tangent to the circumference of a circle or the surface of a cylinder. These directions are crucial in the analysis of residual stresses because materials can exhibit different mechanical behaviours along different crystallographic axes. Also, data is presented for the RD_corr which is the correction applied to the RD data to account for factors that might influence the accuracy of the data.

The sheet designated stress_S1, stress_S2, stress_S3 and stress_S4 shows the neutron experimental conditions used to obtain the residual stresses in the coatings. These include energy of incident neutrons (E), crystallographic miller index (H), energy of the scattered neutrons (Es), lattice spacing (d), doppler broadening (Ed), angular frequency of neutrons (w), structure factor (sq), energy resolution (dE) and time resolution (dt). From these conditions the neutrons deflected, corresponding crystallographic indices and residual stresses can be calculated as shown in the xlsx file.

## 3 Experimental Design, Materials and Methods

### 3.1 Samples and spraying conditions

The HVOF system adopted in this data acquisition is a Sulzer Metco HVOF hybrid DJ-2600, housed at Thermaspray Company in South Africa. In this system, kerosene is used as liquid fuel and is combusted with oxygen to produce a hot gas jet into which the powder particles are radially injected using nitrogen as the carrier gas [[2], [3], [4]]. The Inconel 625 coating was deposited onto four rectangular (100×100×3 mm) 304 stainless steel substrates. The chemical composition of both the Inconel 625 and 304 stainless steel have been presentd in Table 1. Prior to deposition, all substrates were grit blasted with aluminium and degreased. The process parameters were kept constant, whilst the coating thicknesses were varied. The coating thickness of 250 µm, 300 µm, 400 µm, and 500 µm was produced. Table 2 shows the thermal coating parameters employed.

**Table 1:** Chemical composition of Inconel 625 and 304 stainless steel as %wt.

	Ni	Cr	Mo	Fe	Nb+Ta	Ti	Al	Si	S	Mn	Co	C	P
Inconel 625	Balan	21.7	8.8	3.9	3.9	0.23	0.17	0.15	-	0.14	0.08	0.05	-
304 SS	10.5	20	-	Balan	-	-	-	0.75	0.03	2	-	0.08	0.045

**Table 2:** Thermal spray deposition parameters [3,4].

Oxygen flow rate (m^3^/h)	(Kerosene) flow rate (m^3^/h)	Powder feed rate (g/min)	Chamber Pressure (bar)	Stand-off distance (mm)	Nozzle length (mm)
52.39	0.03	80	7.8	375	101.6

### 3.2 Residual stress

The residual stress by neutron diffraction techniques were measured at ANSTO OPAL reactor neutron facility (Australia) using KOWARI stress diffractometer. The measurement in the 304 SS were done through-thickness using wavelength of 1.55Å obtained from Si (400) at the take-off angle of 69°. The γ –Fe (311) peak at 90.6° with gauge volume of 0.2×0.2×20 mm^3^ was utilised during strain measurement in steps of 0.2 mm in two principal directions, in-plane and normal to the surface. Like the other diffraction techniques, the diffracted beam of neutrons behaves according to Bragg's Law enabling the detection of changes in atomic lattice spacing due to stress. The relative changes in spacing are then calibrated using a stress-free material sample to calculate absolute stress values [5,6]. Table 3 shows the coating thickness and their resultant average residual stress values.

**Table 3:** Measured residual stress data of coated samples [[2], [3]].

Coating thickness ( µm)	Average residual stress (MPa)
250	-270.2 ± 7.2
300	-306.1 ± 12.7
400	-355.1 ± 24.8
500	-339.5 ± 40.7

## Limitations

Not applicable.

## Ethics Statement

The authors have read and follow the ethical requirements for publication in Data in Brief and the current work does not involve human subjects, animal experiments, or any data collected from social media platforms.

## CRediT authorship contribution statement

**Oluseyi Philip Oladijo:** Conceptualization, Methodology, Writing – original draft, Validation. **Vladimir Luzin:** Investigation, Resources. **Michael Oluwatosin Bodunrin:** Funding acquisition. **Tien-Chen Jen:** Writing – review & editing. **Resego Phiri:** Writing – review & editing, Data curation.

## Data Availability

HVOF Residual Stress (Original data) (Mendeley Data).
